# Availability and affordability of essential medicines for children in the Western part of Ethiopia: implication for access

**DOI:** 10.1186/s12887-016-0572-3

**Published:** 2016-03-15

**Authors:** Edao Sado, Alemu Sufa

**Affiliations:** Department of Pharmacy, Pharmacoepidemiology and Social Pharmacy Unit, College of Medical and Health Sciences, Wollega University Ethiopia, P.O. Box 395, Nekemte, Ethiopia; Department of Public health, Reproductive Health Unit, College of Medical and Health Sciences, Wollega University Ethiopia, P.O. Box 395, Nekemte, Ethiopia

**Keywords:** Access to medicine, Children, Availability, Affordability, Prices of medicine, East wollega zone, Nekemte town, Ethiopia

## Abstract

**Background:**

Essential medicines (EMs) are those medicines which satisfy the priority health care needs of the population. Although it is a fundamental human right, access to essential medicines has been a big challenge in developing countries particularly for children. WHO recommends assessing the current situations on availability and affordability of EMs as the first step towards enhancing access to them. Therefore, the aim of this study was to assess access to EMs for children based on availability, affordability, and price.

**Methods:**

We adapted the WHO and Health Action International tools to measure availability, affordability, and prices of EMs. We collected data on 22 EMs for children from 15 public to 40 private sectors’ drug outlets in east Wollega zone. Availability was expressed as percentage of drug outlets per sector that stocked surveyed medicines on the day of data collection, and prices were expressed as median price ratio. Affordability was measured as the number of daily wages required for the lowest-paid government unskilled worker (1.04 US $per day) to purchase one standard treatment of an acute condition or treatment for a chronic condition for a month.

**Results:**

The average availability of essential medicines was 43 % at public and 42.8 % at private sectors. Lowest priced medicines were sold at median of 1.18 and 1.54 times their international reference prices (IRP) in the public and private sectors, respectively. Half of these medicines were priced at 0.90 to 1.3 in the public sector and 1.23 to 2.07 in the private sector times their respective IRP. Patient prices were 36 % times higher in the private sector than in the public sector. Medicines were unaffordable for treatment of common conditions prevalent in the zone at both public and private sectors as they cost a day or more days’ wages for the lowest paid government unskilled worker.

**Conclusions:**

Access to EMs to children is hampered by low availability and high price which is unaffordable. Thus, further study on larger scale is critical to identify acute areas for policy interventions such as price and or supply, and to enhance access to EMs to children.

**Electronic supplementary material:**

The online version of this article (doi:10.1186/s12887-016-0572-3) contains supplementary material, which is available to authorized users.

## Background

Essential medicines (EMs), which satisfy the priority health care needs of the population, are backbone of health care and well being of individuals and populations [1–3]. Access to health care including EMs is a fundamental human right [4, 5]. However, access to EMs has been a big challenge particularly in developing countries where more than half of their populations lack access to EMs [6] and majority of them are children [7].

Access to EMs is influenced by many interlinked factors such as the availability of medicines in the health care facilities, availability of sustainable financing and reliable health systems, rational selection and use of medicines, and affordable price [1, 8, 9]. In addition to these factors, they are also hardly found in the health care facilities in the recommended dosage for children [10, 11]. This shows the inaccessibility of EMs for children in the developing countries where majority of child mortality is due to treatable diseases [10, 12]. This is also true for Ethiopia, where more than 60 % of child deaths are due to communicable diseases [13].

In order to escalate the accessibility of EMs for children, World Health Organization (WHO) developed Essential Medicine Lists for children (EMLc) in 2007 and it has also been promoting to formulate medicines in line with child body size through “make medicine child size” initiative [10, 14]. The initiative aims to enhance the accessibility of safe, effective and quality medicines for children by promoting awareness and action through research, regulatory measures and changes in policy [10]. In line with the initiative, measuring the availability and prices of essential medicines in all sectors is a vital step to improve the accessibility of EMs for children [10].

Data on the availability and affordability of EMs help managers and policy makers to develop national policy, regulations and strategies to enhance access to them. However, there are fewer studies which provide these types of data for managers and policy makers. A study conducted on the availability and prices of the WHO’s EMs for children in Guatemala revealed that availability of EMs is less than 50 % in both private and public sectors, and prices for both lower priced medicines and higher priced medicines are higher than the respective international reference prices (IRP) and unaffordable, costing as much of 15 days’ wages [10]. Similar finding is reported from the studies conducted in China [15, 16]. There is also a survey conducted in fourteen central Africa countries which showed poor availability of EMs for children in both private and public sectors, and higher prices with considerable variations [17]. A national study conducted by Abiye and his colleagues in the western part of Ethiopia showed that availability of medicine is almost higher than 50 %, and medicines are sold at average of 0.65 and 0.94 times the IRP in the public and private sectors, respectively [18].

Although limited access to EMs for children is a global problem [19, 20], it is pressing issue in developing countries particularly for Sub-Sahara Africa countries [21]. Beyond that, the extent of the problem in Ethiopia is unknown. To the authors’ best knowledge, the previous study on the availability and affordability of EMs in the western part of Ethiopia [18] only focused on the medicines for adult, and it was also not conducted according to WHO/Health Action International (HAI) methodology. So, there has been no study conducted on the availability and affordability of EMs for children in Ethiopia. Therefore, the purpose of this study was to assess the availability, prices and affordability of EMs for children to determine their accessibility for children.

## Methods

### Study area and design

A drug outlets based cross-sectional study was conducted in east Wollega zone, western part of Ethiopia. Data on the availability and prices of 22 EMs for children were collected in January, 2015 by adapting of the WHO/HAI standardized methodology [22].

### Selection of drug outlets

Ten districts were randomly selected from the seventeen districts found in the east Wollega zone. There were a total of 56 drug outlets found in the ten selected districts surrounding Nekemte town, the capital city of the zone, and 40 drug outlets in the Nekemte town. These drug outlets were stratified into public, private and other (NGO drug outlets) sectors. From public sector, at least one drug outlet per district was randomly selected and included, but one hospital pharmacy, found in the surrounding district, was included purposely according to WHO/HAI recommendation [22]. From private sector, at least two drug outlets per district were randomly selected and included in the study. Private drug outlets were selected at a ratio of 2:1 compared to public drug outlets because the number of private drug outlets is 2–5 times higher than public drug outlets in the selected districts, and private outlets serve as major sources of drugs for the public. However, all drug outlets of other sector found in the surrounding districts and the town were included purposely. We also included all three public drug outlets and one hospital pharmacy found in the town, purposely. Among 40 private outlets found in the town, 22 drug outlets were randomly selected and included in the study.

### Selection of medicines

Twenty three EMs were identified based on the core list of the WHO EMLc specified by the “Better Medicines for Children Project” effort [23] and prevalence of diseases associated with childhood illness in the zone [East Wollega Health Department]. For each surveyed medicine, we collected data on the lowest priced, highest priced (instead of innovator/brand medicines), and its availability. But for antimalaria medicines, vitamin A and Zinc which are free of charge for public at public sector, we checked only their availability.

### Data collection and analysis

We collected data on the availability and patient prices of medicines from 58 drug outlets during January, 2015. Among 58 drug outlets, 15 were from the public sector, 41 were from the private sector and two were from the other sector. Five data collectors were recruited and trained according to WHO/HAI methodology and pretest was conducted in Ghimbi town, as it has close geographic proximity and population with similar socioeconomic status, and similar distribution of drug outlets. The data collectors collected information on availability and price using a standard data collection format specific to the EMs under survey Additional file 1. Then,” we entered data into the pre-programmed MS Excel Workbook provided as part of the WHO/HAI methodology” [22]. Data were double entered, cleared and analysed by using MS Excel Workbook provided by WHO/HAI Management Sciences for Health (MSH) 2012 part I. We presented the results by using tables and bar chart.

Though data were collected from 58 drug outlets, we analysed only the data collected from 55 drug outlets where 15 of them were from the public sector and 40 were from the private sector. We excluded data collected from one private drug outlet because the collected information was incomplete. Two drug outlets from other sector were also excluded as they do not fulfill the WHO/HAI recommendation criteria; the minimum number of drug outlets per sector should be four or greater than four [22].

Among the twenty three surveyed drugs, we included only twenty two drugs in analysis for both public and private sectors (Table 1). We excluded phenobarbitone (Phenobarbital) 20 mg/5 ml elixir from analysis as the information was not yet collected because wrong targeted pack size was used in data collection formats.

**Table 1:** Lists of medicines surveyed in east Wollega zone

No	Name	Strength	Dosage form	Indications
1.	Amoxicillin	125 mg/5 ml	Suspension	Infectious disease
2.	Amoxicillin	250 mg/5 ml	Suspension	Infectious disease
3.	Amoxicillin + Clavulanic Acid	125 mg + 31.25 mg/5 ml	Suspension	Infectious disease
4.	Amoxicillin + Clavulanic Acid	250 mg + 62.5 mg/5 ml	Suspension	Infectious disease
5.	Artesunate^a^	60 mg	Vial	Infectious disease
6.	Artemether + Lumefantrine^a^	20 mg + 120 mg	Dispersible tab.	Infectious disease
7.	Chloramphenicol	1 gm	Vial	Infectious disease
8.	Carbamazepine	100 mg/5 ml	Syrup	Seizure disorder
9.	Ceftriaxone	500 mg	Vial	Infectious disease
10.	Cotrimoxazole	40 mg + 200 mg/5 ml	Suspension	Infectious disease
11.	Diazepam	5 mg/ml	Ampoule	Seizure disorder
12.	Gentamicin	20 mg/2 ml	Ampoule	Infectious disease
13.	Ibuprofen	100 mg/5 ml	Suspension	Pain/inflammation
14.	ORS	To make 500 ml	Powder	Dehydration
15.	Paracetamol	120 mg/5 ml	Syrup	Pain
16.	Paracetamol	125 mg	Suppository	Pain
17.	Penicillin G	1 million IU	Vial	Infectious disease
18.	Procaine Penicillin G	4 million IU	Vial	Infectious disease
19.	Salbutamol	100 mcg/dose	Inhaler	Asthma
20.	Vitamin A^a^	50,000 units	Capsule	Xerophthalmia
21.	Zinc Phosphate^a^	20 mg	Dispersible tab	Dehydration
22.	Procaine Penicillin G	4 million IU	Vial	Infectious disease

^a^Medicines free of charge to the public in the public sector

*ORS* oral rehydration salt

### Measuring availability and affordability of medicines

We used IRP of 2014 given by Management Sciences for Health (MSH) to facilitate national and international comparisons. The MSH reference prices are the medians of recent procurement prices offered for generic products by not-for-profit suppliers to developing countries [24]. For cross-country comparisons purpose, we expressed prices as median price ratios (MPR). MPR is ratio of median local unit price relative to IRP [10]:

$$ \mathrm{M}\mathrm{P}\mathrm{R}=\frac{\mathrm{Median}.\mathrm{local}.\mathrm{unit}.\mathrm{price}}{\mathrm{International}.\mathrm{reference}.\mathrm{unit}.\mathrm{price}} $$

We calculated MPR only for medicines with price data obtained from at least 4 drug outlets according to WHO/HAI recommendation. We used 1 US$ = 19.6758 Ethiopian Birr exchange rate to calculate MPR, and it was commercial buying rate obtained from www.combanketh.et/currencyrate on the first day of data collection [25].

We measured availability by physical presence of EMs in the drug outlets on single visit during data collections. We expressed it as percentage of sampled drug outlets that have a particular EMs [26].

We assessed affordability for a standard treatment of top ten prevalent diseases in the childhood by comparing the total price of medicine at a standard dose according to Ethiopian standard treatment guideline for pediatrics to the daily wage of the lowest paid government unskilled employee at 20.5 Ethiopian birr (1.04 US $) per day at the time of data collection. The cost of medicine for a full course of therapy for acute diseases and a 30-days’ supply of medicines for chronic diseases was calculated and changed to the day wage. Even though it is difficult to assess the real affordability of the medicine, we categorized as a medicine affordable “if it costs less than a day wage and unaffordable if it costs a day wage or more than a day wages” [10].

### Ethical considerations

The study protocol was reviewed and approved by Institutional Research Review Committee of College of Medical and Health Sciences, Wollega University. The owners of drug outlets who participated were informed of the aims of study prior to participation, and a verbal consent was sought from each participated owner of drug outlet after explaining his/her right not to participate into the study. They were assured of confidentiality on the issues related to the business secret of premises by avoiding identifiers from the data collection tools.

## Results

### Availability of medicines on the day of data collection

The results as shown in Table 2 revealed that the availability of lowest priced individual medicines varied by type of medicine and sector. It was found that average availability of the highest priced medicines was 1.2 % (range 0–4.5 %) and 43.0 % (range 10.7–75 %) for the lowest priced medicines in the public sector. Average availability in the private sector was 7.4 % (range 0–18.3 %) for the highest priced medicines and 42.8 % (range 6.5–77.1 %) for lowest priced medicines.

**Table 2:** Average availability of individual lowest priced medicines in the public and private sectors

Name of medicine	Percentage of outlets where medicine was found	
	Public sector (*n* = 15 outlets)	Private sector (*n* = 40 outlets)
Amoxicillin Suspension 125 mg	80 %	90 %
Amoxicillin Suspension 250 mg	60 %	80 %
Amoxicillin- Clav^a^ Suspension 125 mg	66.7 %	47.5 %
Amoxicillin- Clav^a^ Suspension 250 mg	13.3 %	40 %
Artesunate 60 mg vial	26.7 %	0.0 %
Artemether + Lumefantrine 20 mg + 120 mg disp. tab	66.7 %	0.0 %
Chloramphenicol 1gm vial	46.7 %	20 %
Carbamazepine 100 mg/5 ml syrup	0.0 %	0 %
Ceftriaxone 500 mg vial	26.7 %	22.5 %
Cotrimoxazole 40 mg + 200 mg/5 ml suspension	100 %	90 %
Diazepam 5 mg/ml ampoule	0.0 %	0.0 %
Gentamicin 20 mg/2 ml ampoule	0.0 %	0.0 %
Ibuprofen 100 mg/5 ml Suspension	0.0 %	0.0 %
ORS to make 500 ml soln.	0.0 %	2.5 %
ORS to make 1000 ml soln.	40 %	87.5 %
Paracetamol 120 mg/5 ml Syrup	93.3 %	80 %
Paracetamol 125 mg Suppository	93.3 %	87.5 %
Penicillin G 1million IU vial	46.7 %	12.5 %
Procaine Penicillin G 4 million IU vial	40 %	57.5 %
Salbutamol 100 mcg/dose inhaler	46.7 %	52.5 %
Vitamin A 50,000 units Cap.	20 %	0.0 %
Zinc Phosphate	66.7 %	0.0 %

Clav^a^: − Clavulanic acid/Clavunate

In the public drug outlets, generic medicines were the predominant product type available with 96 % of medicines found as generics. Although vitamin A, zinc phosphate and antimalaria medicines are expected to be available in the public sector only, some of them such as artesunate (26.7 %) and vitamin A (20 %) had low availability. Carbamazepine 100 mg/5 ml syrup, diazepam 5 mg/ml ampoule, gentamicin 20 mg/2 ml ampoule and ibuprofen 100 mg/5 ml suspension were not found in any drug outlets in both public and private sectors. The average availability of individual lowest price medicines in both public and private sectors was shown in Table 2.

### Costs of medicines in public and private sectors

To assess price variation of individual medicine across sectors, we calculated MPR of 13 (*n* = 13) lowest priced medicines. As shown in Table 3, MPR for lowest price medicines were found to be 1.18 times their IRP in the public sector. MPR for patient prices ranged from 0.58 to 2.86 times the IRP in the public sector for paracetamol suppository and penicillin G injection respectively. Half of lowest priced medicines were priced at 0.90 (25^th^ percentile) to 1.3 (75^th^ percentile) times their IRP in the public sector, showing small variation within sector.

**Table 3:** Median price ratios of thirteen lowest priced medicines in the public and private sectors (*n* = 13)

Medicine name	Lowest price medicines MPR (25^th^-75^th^ percentile)	
	Public sector	Private sector
Amoxicillin Suspension 125 mg	1.24(1.14–1.240)	2.07(1.87–2.07)
Amoxicillin Suspension 250 mg	1.47(1.34–1.54)	1.67(1.67– 1.89)
Amoxicillin- Clav Suspension 125 mg	0.62(0.48–0.98)	1.52(1.42–1.52)
Amoxicillin- Clav Suspension 250 mg	N/A	1.05(0.91–1.11)
Chloramphenicol 1 gm vial	0.93(0.86–1.10)	1.10(1.10–1.10)
Ceftriaxone 500 mg vial	0.95(0.89 – 1.03)	5.02(4.47–5.02)
Cotrimoxazole 40 mg + 200 mg/5 ml Suspension	1.20(1.20–1.40)	1.49(1.40–1.59)
ORS to make 1000 ml soln.	1.53(1.15– 1.93)	2.55(1.78–2.55)
Paracetamol 120 mg/5 ml Syrup	1.17(0.90–1.32)	1.23(0.87–1.47)
Paracetamol 125 mg Suppository	0.58(0.49–0.59)	0.58(0.58–0.93)
Penicillin G 1 million IU	2.86(2.05–3.31)	3.15(2.84–3.15)
Procaine Penicillin G 4million IU	1.27(1.14–1.41)	1.84(1.63 – 2.04)
Salbutamol 100 mcg/dose inhaler	0.80(0.80–1.00)	1.54(1.16– 10.27)

In the private sector, MPR for lowest priced medicines were found to be 1.54 times their IRP, and patient prices were ranged from 0.58 to 5.02 times the IRP for paracetamol suppository and ceftriaxone injection respectively. Half of the lowest priced medicines were priced at 1.23 (25^th^ percentile) to 2.07 (75^th^ percentile) times the IRP in the private sector, showing moderate variation in medicine price ratios across individual lowest priced medicines.

Highest priced medicines were found in less than four drug outlets of public sector. So, we did not calculate their MPR. But in the private sector, their MPR were 3.01 times IRP.

### Comparison of costs in the public and private sectors

**Fig. 1:**
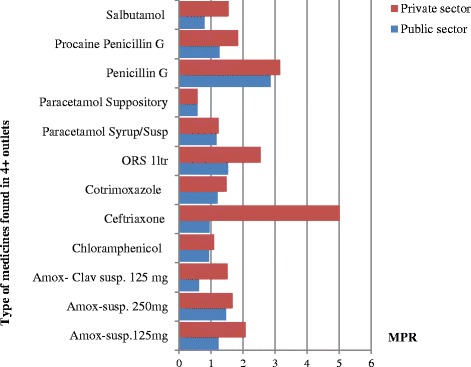
Comparison of MPR of lowest price medicines found in atleast four drug outlets in public and private sectors

To compare patient prices across sectors, we used twelve (*n* = 12) lowest priced medicines found in at least four drug outlets in both public and private sectors, and we calculated their MPR as depicted in Fig. 1. Except for paracetamol suppository which had similar MPR in both public and private sectors, MPR were moderately higher in the private sector compared to the public sector but substantially higher for ceftriaxone injection. Median price ratios for these medicines were 1.18 and 1.61 in the public and private sector respectively; patient prices were 36 % times higher in the private sector than in the public sector.

### Affordability of medicines for standard treatment regimens

As shown in Table 4, 70 % (7/10) of treatments of common childhood diseases prevalent in the zone with standard treatment [27] were unaffordable, as they cost a day’s wage or more days’ wages in both sectors. The unaffordability of lowest priced medicines in the public sector varies from 1.5 to 8.7 days’ wages. Treatments of typhoid fever with chloramphenicol 1gm (8.7 days’ wages) and infections due to susceptible organism with ceftriaxone 500 mg (5.8 days wages) cost more than 5 days’ wages, and they were the most unaffordable standard treatments in the public sector.

**Table 4:** Affordability: number of days' wage of lowest paid unskilled government worker makes to purchase standard treatments

Conditions	Drug name, strength, dosage form	Treatment schedule	Days wages to pay for treatment	
			Public sector	Private sector
Typhoid fever	Chloramphenicol 1gm vial	25 mg/kg * 14.5 kg every 6 hours P.O. for 14 days = 20300 mg = 20.3gm 21vial [26].	8.7	10.2
Asthma	Salbutamol 100 mcg/dose inhaler	1 inhaler of 200doses [27].	1.5	2.9
Dysentery	Cotrimoxazole 40 mg + 200 mg/5 ml Suspension	24 mg/kg * 14.5 kg BID for 5 days = 3480 mg.72.5 ml total for five days [27].	0.4	0.5
Severe Pneumonia	Penicillin G 1 million IU vial	50 000 units/kg ^a^14.5 kg IV every 4 hours for at least 3 days = 13.05 millions of IU = 14vial for three days [27].	3.1	3.4
Acute otitis media	Amoxicillin 250 mg/5 ml Suspension	250 mg/5 ml P.O. TID for 10 days for children above 6 years of age = 150 ml [27].	1.6	1.8
Acute rhinosinusitis	Amoxicillin- Clav 156 mg/5 ml	156 mg/5 ml P.O. TID for 10dys = 150 ml [27].	2.5	5.9
Acute bacterial tonsillitis	Amoxicillin- Clav 156 mg/5 ml	156 mg/5 ml P.O. BID for ten days = 150 ml [27].	2.5	5.9
Dehydration	ORS to make 1litre	Moderate dehydration 75 ml/kg * 14.5 = 1087.5 [27].	0.3	0.5
Infections due to susceptible organism	Ceftriaxone 500 mg vial	Child under 50 kg maximum 1gm for 7 days [27].	5.8	30.7
Pain/managenment	Paracetamol 125 mg/5 ml Suspension	5 year old child: 15 mg/kg*14.5 kg*4*3 = 104.4 ml [27].	0.8	0.8

*Weight of average 5 year old child in Ethiopia = 14.5 kg [13, 34]

*P.O*. per oral, *BID* two times per a day, *TID* three times per a day

As shown in Table 4, the unaffordability of the lowest priced medicines varies from 1.8 to 30.7 days’ wages in the private sector. The most unaffordable standard treatments were treatment of typhoid fever with chloramphenicol1gm (10.2 days’ wages) and treatment of infections due to susceptible organism with ceftriaxone 500 mg (30.7 days’ wages).

## Discussions

The findings of present study suggest that availability of children’s EMs is below 50 % in both public sector and private sector for both types of surveyed category of medicines. The average availability of lowest priced medicines for children is 43.0 % in the public and 42.8 % in the private sectors. Because of the general incomparability of survey results (due to variation in medicine pricing policy, methodology, types of prevalent disease, and medicine supply systems), it is difficult to make a comparative analysis of medicines availability. However, these findings are consistent with findings of study conducted by Anson et al. [10] in Guatemala which reported 46 % in public sector and 35 % in private sector. In contrast to the study conducted by Wang et al. [16] in China, this finding showed higher availability of lowest priced medicines in both public and private sectors, but it showed lower availability of highest priced medicines in both public and private sectors. The study also revealed that availability of medicines was higher in the public sector than in the private sector. This finding is also consistent with findings of studies conducted by Anson et al. [10] and Wang et al. [16].

When we compare availability of medicines for children and for adults (or for overall population) perspective, the finding is lower than the finding of Abiye and his colleagues study in the western part of Ethiopia for public drug outlets [18]. But it is consistent for private sector and higher for public sector compared with findings of study conducted by Babar et al. [27] in Malaysia. It is also similar for public sector and lower for private sector from the findings reported by Bazargani et al. [29]. In opposite to the current findings, availability of medicines was higher in the private sector as compared to the public sector for mixed or general populations [28, 29]. The low availability of medicines in the formulations preferable for use in children may limit access of medicines to children. To tackle this problem, health care professionals particularly pharmacists and nurses calculate the dose from adult dosage. This calculation may lead to incorrect dose use which might cause adverse drug effect [30].

EMs used for the treatment of chronic diseases in children were hardly found. This very low availability of medicines for treatment of chronic diseases in children consistent with government policy which is more focuses on the prevention rather than treatment [31]. Medicines offered free of charge from public sector are not available in any private sector and even their availability in public sector is low which may cause the inaccessibility of EMs for children. Though the most common causes of child morbidity and mortality are infectious diseases [13, 31], medicines used to treat common infectious disease in children like gentamicin is also not available in any drug outlets in both public and private sectors. This unavailability of common medicines for treatment of infectious diseases in the children might be due to lack of focuses from the government policy.

Like adult medications, the availability of child specific lowest priced medicines far exceeded that of highest priced medicines across all drug outlets in the public and private sectors. Highest priced medicines are unavailable in the 98.8 % of public sector drug outlets. This may be due to generic procurement promotion in the public drug outlets. The availability of lowest price medicines in the drug outlets ranges from 5 to 45 % in both public and private sectors. This is consistent with findings of study conducted by Robertson et al. [17] and his colleagues even though the perspective of study is not same.

The study also revealed that lowest priced medicines for children in both public and private sectors were sold at higher price than IRP. In the public sector, they are sold at 1.18 times their IRP and 1.54 times their IRP in the private sector. This finding is similar with the study conducted on the prices, availability and affordability of medicine in China [11] and findings of a study conducted by Cameron et al. (2008) and his colleague [32]. There was a notable variability in prices across drug outlets in private sector. This finding is consistent with study conducted on the availability, prices and affordability of essential medicines in Haiti [26]. The variability of price across the drug outlets in private sector might be the result of high market competition.

Lowest priced medicines are unaffordable for 70 % of standard treatments of prevalent infectious diseases in both sectors as they cost a day’s wage or more days’ wages for lowest paid government employee. However, the extent they cost varies between the public and private drug outlets. This finding is consistent with the findings from study done on the availability, prices and affordability of the World Health Organization’s essential medicines for children in Guatemala [10]. These costs do not include the costs of consultation and diagnostic tests, so that families who need medicines for more than one child may be confronted with more costs and extra days’ wages. These findings are inconsistent with other studies of affordability of adult medicines which showed unaffordability of chronic medicines rather than drugs for infectious treatment for low income populations [10, 29] and consistent with study of affordability in the Haiti [26].

Although Ethiopia achieved Millennium Development Goal for reducing child mortality rate 2 years ahead of 2015 [33], the findings from this study suggest that accessibility of EMs for children is still low. Therefore, there is a need of improving the access to EMs for children which will help the country to achieve the global strategy for every child as part of the Sustainable Development Goals.

### Limitation of the study

This study did not assess the medicine procurement prices and it was also conducted in the one zone due to logistical constraints.

## Conclusion

This study was conducted to assess access to essential medicines for children based on their availability, price, and affordability. It has shown that availability of EMs for children use was below the recommended average in both public and private sectors. But the unavailability of EMs offered free of charge from public sector was pressing problem. Medicines were sold at higher price of IRPs and were unaffordable for people with low income in both public and private sectors.

The findings of this study suggest that access to EMs to children is hampered by low availability and high price which is unaffordability. Thus, further study should be conducted on larger scale by including medicines procurement price to identify acute areas for policy interventions such as price and or supply to enhance access to EMs to children.

## Availability of data and materials

The datasets supporting the conclusions of this article is included within the manuscript and supporting information.

## Additional file

Additional file 1: Medicine Price Data Collection Form. (XLSX 16 kb)

## Abbreviations

EMLc: essential medicine lists for children
EMs: essential medicines
HAI: health action international
IRP: international reference prices
MPR: median price ratios
MSH: management sciences for health
WHO: World Health Organization
